# Caregiving burden and special needs of parents in the care of their short-statured children – a qualitative approach

**DOI:** 10.3389/fendo.2023.1093983

**Published:** 2023-03-17

**Authors:** Lea Lackner, Julia Hannah Quitmann, Stefanie Witt

**Affiliations:** Department of Medical Psychology, Center for Psychosocial Medicine, University Medical Center Hamburg-Eppendorf, Hamburg, Germany

**Keywords:** health-related quality of life, short stature, caregiving burden, children and parents, isolated growth hormone deficiency, idiopathic short stature

## Abstract

**Purpose:**

To explore caregiving burden, health-related quality of life (HRQOL), stress, and individual resources of parents in the care of children with isolated growth hormone deficiency (IGHD) or idiopathic short stature (ISS).

**Methods:**

Focused interview analysis of previously, within the *Quality of Life in Short Stature Youth* (*QoLISSY*) project, conducted structured focus group discussions (n=7) with parents (n=33) of children with IGHD/ISS aged 4 to 18 years were performed.

**Results:**

26 out of the 33 parents reported mental stress due to their child’s growth disorder. Social pressure and stigmatization were also mentioned as being demanding. Some parents reported having trouble with human growth hormone (hGH) treatment. Several parents wished for parent support groups with other like-minded parents of short-statured children.

**Conclusion:**

For physicians, it is essential to understand the parents’ caregiving burden, stress, and individual resources in caring for IGHD/ISS children. If an impaired HRQOL is detected, psychological intervention for these parents may be scheduled, and coping mechanisms may be discussed. Furthermore, it seems essential for parents to be educated by their healthcare provider about the possible side effects of hGH treatment or to know where to find evidence-based information about it.

## 1 Introduction

Short stature is a chronic health condition defined as a height below the 3rd percentile based on sex, age, and population. Alternatively, the standard deviation score (SDS) can be used to define short stature (1). The causes of short stature can be both a norm variant of growth and a primary or secondary acquired pathological disorder or idiopathic (2). The diagnosis of short stature includes various anthropometric, biochemical, and radiological assessments, such as nutritional and hormonal assessment and an estimation of the children’s bone age (3).

However, isolated growth hormone deficiency (IGHD) and idiopathic short stature (ISS) are most common and, therefore, especially relevant for everyday clinical practice (4).

IGHD is diagnosed when short stature is due to substantiated growth hormone (GH) deficiency. Characteristically, IGHD is defined as a growth rate below the 25th percentile and evidence of retardation of bone age (5). Insulin-like growth factor I (IGF-1) and IGF binding protein 3 (IGF-BP-3) below -2 SD indicate IGHD. Clarification is usually conducted using two GH stimulation tests (6); a nocturnal GH secretion profile can be obtained as an alternative to a stimulation test. Without human GH (hGH) treatment, affected individuals achieve a mean final body height of 4.7 SD below the population mean (7). The gain in body height with hGH treatment is an average of 1.5 to 2.0 SD and, in extreme cases, up to 3.5 SD (7).

ISS refers to a heterogeneous group of patients without a known cause for their short stature. It is a diagnosis that requires complex diagnostics and, if necessary, genetic testing to exclude other (rare) causes (8). ISS includes familial short stature, where body height in adulthood is usually within the familial target height range (9).

IGHD and ISS are not life-threatening diagnoses for children but can still lead to an impaired health-related quality of life (HRQOL) and effect the well-being of the short-statured children, their caregivers, and even the whole family (4, 10–12).

HRQOL is a multidimensional concept of physical, psychological, and social dimensions, including general perceptions of life satisfaction in the context of health (13). The development nd well-being of children with chronic health conditions are directly related to the HRQOL of their parents (14, 15). Thus, exploring and understanding potential reasons for an impaired HRQOL and the caregiving burden that may arise from the child’s health condition is essential.

A child’s chronic health condition requires a future-oriented approach, which aims to teach the children to be independent and successfully integrate the treatment into daily life (16). Studies show that the better adaption of the condition to the family’s everyday life, the less burdensome the disease is perceived and classified by the affected family member (17, 18). The personal responsibility of those involved is fundamental and directly influences the medical prognosis (19). Both parents are affected by the child’s chronic health condition, although mothers are often more involved in caregiving and mainly report to be the primary caregiver (20–22). Mothers consider themselves more psychosocially burdened (23). Mothers and fathers experience disadvantages compared to parents of healthy children regarding personal needs (24, 25). For parents, a chronic health condition of their child results in challenges regarding the balance of care and support on the one hand and the support of independence on the other hand (26, 27).

Chronic conditions in children and adolescents are often associated with significantly poorer HRQOL and a higher caregiving burden on the children’s parents (24, 28–31). The family’s dealing with the health condition depends on the experienced caregiver burden and influences the affected child’s assessment of the HRQOL and treatment adherence (32–34).

Therefore, we aimed to describe the parental caregiving burdens, HRQOL, stress, and individual resources in the care of IGHD/ISS children to address the unmet needs of parents of short-statured children and improve the HRQOL of all family members. The better parents deal with their child’s chronic condition, the better they can meet the child’s development tasks and their own needs (27).

## 2 Materials and methods

### 2.1 QoLISSY project

This analysis used data from the *Quality of Life in Short Stature Youth* (*QoLISSY*) project. This project was a multicenter study in five European countries (Sweden, Spain, France, the United Kingdom, and Germany). The *QoLISSY* study aimed to develop and establish a cross‐cultural condition-specific HRQOL instrument for IGHD/ISS children and adolescents. The QoLISSY questionnaire contains self-reports for children ages 8 to 18 and proxy-reports for children ages 4 to 18 (35).

The project was divided into three phases: 1. focus group discussions, 2. pilot testing and cognitive debriefing, and 3. psychometric testing (field and retest). The current analysis used the German statements from the parent’s focus group discussions. The regional ethics board approved the study before it started (PV3184). A regional ethic board achieved an additional ethic statement for the re-analysis of this data (LPEK-0579a).

### 2.2 Focus groups

As part of the QoLISSY study, seven structured focus group discussions were conducted with 33 parents of children with IGHD/ISS aged 4 to 18. Parents were recruited through the cooperating clinical centers in Bonn, Erlangen, Hamburg, and Munich. Participants received verbal and written information about the study and had to sign the informed consent before focus group participation. Two trained moderators led the focus groups and followed a semi-structured interview guideline. The focus groups were tape-recorded after receiving the informed consent of the participants (4).

We included parents for interview participation if they met the following inclusion criteria (1): parents of a child with a confirmed diagnosis of IGHD or ISS, independently of treatment status (2), parents of a child aged between 4 and 18 years, and (3) sufficient German language skills to participate in an interview. Parents were excluded if other health conditions of the child were the focus of attention. No additional clinical data were collected.

### 2.3 Data analysis

The interviews were transcribed verbatim all names of the participants and names mentioned by the interviewees were pseudonymized with either letters or names. Based on the interview data, a computer-assisted focused interview analysis (36) was conducted using MaxQDA-Software (MaxQDA 2020). Categories for the coding guide were defined deductively and inductively. The deductive categories and their definitions are based on the interview guideline and a previously performed systematic review (submitted). In addition, inductive main categories and subcategories were added based on the qualitative data.

After the coding process, a reliability check of the focus group statements was conducted to ensure that the results were reproducible. A second coder (SW) coded 20% (n = 2) of the focus groups. An agreement of a minimum of 70% was set previously as the lowest limit. After the first run, there was a 65% intercoder agreement. By discussing difficult sections and then optimizing the coding guideline, we achieved an intercoder agreement of 81%.

## 3 Results

### 3.1 Sample description

A total of 33 parents participated, having at least one child diagnosed with IGHD or ISS - three parents had two children with growth problems, including one mother with identical twins. The participating parents reported about 36 children and adolescents with IGHD or ISS; 26 were male, and ten were female. Three of the 33 parents participated as parent pairs, and 27 participated without their partners (**Table 1**).

**Table 1:** Sample size of parents divided into their children’s age groups, and the children’s diagnosis.

Childrens’ age groups	4-7 years		8-12 years		13-18 years		Total
*Diagnosis*	IGHD	ISS	IGHD	ISS	IGHD	ISS	
*participating mothers*	4	4	4	3	6	7	28
*participating fathers*	1	–	1	–	2	1	5
*total amount of parents*	5	4	5	3	8	8	33

### 3.2 Category system

Five hundred and nine growth-related statements from parents with IGHD/ISS children were coded, resulting in eight main categories related to parents’ caregiving burdens, HRQOL, stress, and special needs due to the child’s short stature (1): *social problems, (2) mental stress, (3) everyday life, (4) growth hormone treatment, (5) special support, (6) future worries, (7) special needs*, and *(8) individual resources.*

#### 3.2.1 Social problems

The category *social problems* includes statements about parental stress from social situations, structures, and relationships due to the child’s growth disorder. These problems mainly cover stigmatization and social pressure some parents experienced throughout their child’s short stature. Statements about social problems related to hGH treatment were coded into the *growth hormone treatment* category.

Parents mainly reported problems due to their child’s growth disorder depending on concrete social situations, structures, and relationships. They felt stressed about comparing their child to peers of the same age in size and skills (n=11; 33%). Some interviewees, for example, found it upsetting that their child was only able to ride the walking bike at the age of five, while other children could already ride the bicycle by then. Other parents mentioned their child’s shorter body height compared to their peers as challenging (n=11; 33%).

*“And when my son did not really grow along with his friends of the same age, that has been depressing me for all these years.”* (Mother of an 18-year-old adolescent with IGHD)

Some parents (n=6; 18%) also distinguish between boys and girls. From their point of view, girls with a growth disorder had a much easier time in daily life than boys, which also made interacting with their social environment easier. One mother said that society expects men to be taller than women.

Parents also reported getting stigmatized because of their child’s short stature, and they mentioned the ignorance of society about short stature. For example, some interviewees (n=7; 21%) outlined that other people thought their child was short because the parents did not provide sufficient food for their child. Some parents (n=9; 27%) mentioned that social standards are demanding.

*“But that’s exactly how I felt, I felt like I had a bad child because she didn’t fit the standard. In society, much emphasis is placed on the standard, and this is quite awful.”* (Mother of a 7-year-old child, ISS)

In some cases, the social environment tried to force the parents of short-statured children to act. Parents (n=7; 21%) reported that other people tried to advise them on what would be best to do. Few parents (n=4; 12%) reported having no social problems, mainly because their surroundings ignored the topic.

#### 3.2.2 Mental stress

Statements were coded into the category *mental stress* when they addressed parents’ mental stress due to their child’s growth problems. If the reported problems were influenced or caused by the social environment, these statements were coded into the category *social problems.*

Almost all the interviewees mentioned their psychological well-being getting affected by their child’s growth problem (n=26; 79%). Often other people’s reactions and behavior toward the parents’ child were the cause for them feeling frustrated. Parents experienced frustration because their children were treated according to height and not age (n=11; 33%).

Parents also reported that they witnessed or heard their children being bullied by peers resulting in isolation and feelings of rejection (n=11; 33%). These experiences negatively affected the parents’ well-being and mental health. Some parents stated that they felt excluded from society because of the growth disorder of their children (n=7; 21%).

*“And now I just felt that this hurts me in part, because for me, what I experience with my daughter is really an enrichment, actually. It really makes my life more beautiful. But often it is not seen, and I notice over and over again that one point with us is really also loneliness. […]. That we lead such a different life. That she very often notices that she is not taken seriously.”* (Mother of a 12-year-old child, ISS)

Some parents felt helpless because they could not permanently protect their children from challenging situations concerning their short stature (n=11; 33%).

*“When I see that she suffers [from her short stature], when she is always asked about it. And at some point you no longer know how you can help her […].”* (Mother of a 13-year-old adolescent, ISS)

Children’s body height was another concern influencing parents’ psychological well-being. Parents mentioned they struggled to determine their child’s estimated future height (n=12; 36%). Some parents mentioned setbacks in gaining height as a problem for their children and themselves. Sometimes the parents had the impression they were more concerned about the child’s height than the children themselves.

Another aspect mentioned by the participating parents was problems within the family (n=8; 24%). Parents described the children’s grandparents having problems accepting their grandchildren’s growth disorder. From the parents’ perspective, some grandparents were ashamed of their grandchildren’s short stature; others accused the parent’s children of overrating the growth problem. In addition, some parents themselves felt bad because they initially underestimated the growth disorder.

*“I also thought that [his height] is normal and you can’t speed it up, and he has to live with it, and the more it’s discussed, the worse it gets for him. Because he then also looks for excuses because he is so small. And that’s why we tended to ignore it until it was no longer possible to ignore.”* (Mother of a 15-year-old adolescent, IGHD)

Additionally, getting a final diagnosis was accompanied by good feelings for most parents, who felt relieved after receiving their child’s diagnosis (n=29; 88%).

#### 3.2.3 Everyday life

The category *everyday life* includes statements about everyday life in the care of short-statured children. Assertions mainly concerned the domains of school, leisure activities, and the home environment, but also physical restrictions on behalf of the parents. If parents reported what special support they had provided for their child in everyday life, this statement was coded into the category *special support.*

Parents mentioned everyday situations from school or kindergarten, especially regarding the contact with professionals, e.g., teachers and pedagogues. Some parents talked to their child’s teacher about the growth disorder and reported having no problems at school (n=6; 18%). Others mentioned having trouble, especially at school, because of a lack of understanding from teachers and peers (n=8; 24%).

*“So I think that [clarification about the growth disorder] is actually the job of the teachers or educators. But they don’t dare to do it, because they don’t know anything about this topic; they are simply afraid of it.”* (Mother of a 10-year-old child with IGHD)

Some parents emphasized that their children had disadvantages in physical education (PE) classes because of their short stature (n=14; 42%). For many parents, this meant they had to mentally support their children intensively when they returned disappointed from PE classes. Parents reported being disappointed because their child had disadvantages because of their short stature in the evaluation of PE, for example, in the high jump.

Parents mentioned shopping trips with their short-statured children (n=17, 52%). Especially problems with buying clothes and bicycles were discussed. Pants were too long, shoes too wide, and many clothes that would fit were inappropriate in style. For these reasons, several interviewees stated that shopping trips are problematic and time-consuming. One mother recounted that she had to shorten all the pants she bought for her short-statured son.

*“Yeah, so that’s all kind of…. When I think about buying the bike. Bikes are ridden by everyone now, but you’ll get them in her size only in pink, which is absolutely babyish. […]. These are such things … these are problems.”* (Mother of a 13-year-old adolescent, IGHD)

Other parents reported that many playgrounds were not designed for small children (n=12, 36%). Often the children would encounter limitations when playing, which was frustrating and burdensome for some parents to watch. One mother mentioned feeling bad because her daughter was too weak to ride a tricycle.

One topic that was not explicitly asked about but raised by a mother was physical limitations. Due to back problems, she could not meet all the demands of everyday life caused by her daughter’s short stature. For example, she could not lift her daughter to have a better view of the zoo animals.

#### 3.2.4 Growth hormone treatment

The category *growth hormone treatment* covers all kinds of parental experiences with hGH treatment, like concerns and stresses, but also positive experiences. Many interviewees’ children were treated with hGH, so parents’ viewpoints regarding hGH therapy were discussed in detail.

Some of the parents whose children were treated with hGH mentioned they were concerned about the side effects of the treatment (n=5; 15%). For example, parents suspected that it could affect the whole body of their child. One mother was afraid of her child getting edema from hGH treatment.

*“For me, for example, it was incredibly horrifying because there was also something about water retention, which can also take place in the head, and then I thought, “Wow, I wouldn’t want to have such a water head child.”* (Mother of a 7-year-old child, ISS)

Many parents reported that the potential side effects did frighten them during decision-making for or against the hGH treatment (n=10; 30%). One father mentioned that he decided for hGH treatment because he wanted to spare his son disadvantages in future life due to his short stature.

Some parents whose children got hGH treatment said, they had difficulties administering the hGH by injection to their children (n=7; 21%). For example, many children feared the injection, which was a problem for the parents because most had to inject their children themselves.

*“But actually, it was really the fear - “Oh God, I still have to inject her.” And then it started, and then you really had to hold her. I had to inject her for weeks [in the clinic], because I couldn’t do it on my own. So the fear of doing it myself, not myself, but in general, no matter who did it. She really resisted.”* (Mother of a 12-year-old child, IGDH)

A few parents mentioned that hGH’s application improved after a while, and their children were more relaxed about the injection. One mother stated that special child-friendly injections made it more fun and straightforward for her child and, at the same time, for herself.

Another topic focused on organizational issues (n=6; 18%). Parents mentioned they had issues going on vacation because the hGH injections need to be cooled all the time. Also, class trips were mentioned as being a problem due to the treatment. One single mother said she had organizational issues when she wanted to go out on a date.

*“So for me it was a problem with the treatment because I’m a single parent, and if I wanted to do something on the weekend, I couldn’t get a normal babysitter, but I always needed a nurse to take over the injection. Now he does it himself, so the issue is settled, but in the beginning, it was bad. […].”* (Mother of a 13-year-old adolescent, IGHD)

One-third of the interviewees whos children were treated with hGH, raised the issue of the pediatrician’s disease handling (n=7; 21%). Many were unsatisfied because their pediatrician trivialized their child’s growth disorder and discouraged the parents from starting with hGH treatment. Some pediatricians even approached the parents with prejudice because the child was short. For example, parents were asked about the abuse of drugs or alcohol while pregnant.

Beyond that, parents mentioned they were happy with hGH treatment (n=5; 15%). Some reported that giving injections became routine, and they were satisfied with their child’s growth success and future prognosis. Parents also mentioned that the hGH treatment got simplified once their child could inject the hGH independently.

#### 3.2.5 Special support

Statements dealing with special care due to the children’s short stature were coded into the category *special support.* The code was assigned when, for example, parents mentioned how they support or treat their short-statured children in a certain way because of their body height, including deliberate “normal” treatment.

Parents’ perception of the need to offer special support to their short-statured child showed an inconsistent picture. On the one hand, parents reported intensive support (n=16; 48%); on the other hand, they reported treating their short-statured children the same way as, for example, healthy siblings (n=11; 33%).

Approximately half of the parents gave social-emotional support to their short-statured children, mainly because of their children’s experiences of being bullied at school resulting in the need for intensive emotional support (n=16; 48%). Parents also mentioned that they strengthen their children’s self-esteem.

*“Yes, you build them up again and again like “You’re strong, you can do it” or “You’re great” or that you also praise small things that don’t matter so much to others that you would praise that.”* (Mother of a 7-year-old child, IGHD)

One mother reported that she only wears flat shoes because her son does not like his mother to be taller than him.

Some parents mentioned treating their short-statured children differently than their siblings (n=14; 42%). For example, they tended to be more overprotective and helpful. One mother said she often helped her short-statured daughter with tasks because otherwise, it would take too much time if her daughter would do it herself.

*“As a mother, you sometimes have the tendency, or I have actually had it, to protect the smaller child a bit against the bigger child because of its short stature.”* (Mother of a 14-year-old adolescent, IGHD)

Other parents reported no difference in treating their short-statured children because they felt that the most regular treatment would help their children prepare for future life (n=11; 33%). For example, some parents said they would hardly discuss the topic of short stature so their children would not feel different from others.

#### 3.2.6 Future worries

Parents’ worries due to the future perspective of their short-statured children were coded into the category *future worries*. Parents focused on their children’s career prospects but also on their later private life and starting a family. Several parents were concerned about their children’s success in these areas of life.

Parents reported worrying about how and whether their children will cope with limitations and restrictions caused by their short stature later in life (n=6; 18%). For example, one mother mentioned she was frightened her short-statured child would not be able to keep up with the others regarding body height and skills. Parents also mentioned that they were concerned their children would not develop properly because they would consistently be underestimated and treated regarding their height and not their age. From the parents’ perspective, short-statured children do not get the opportunity to gain much independence.

*“[His brother] always relieves everything from him, and that is not an advantage for such small children, because they have to develop and do their own thing.”* (Mother of a 7-year-old, IGHD)

Parents reported also being concerned about their children’s future personal life and mentioned that finding a love-mate might be difficult for short-statured children, especially boys (n=4; 12%). Another aspect concerns the independence of short-statured children, and parents mentioned concerns about short-statured children’s possibility of getting a driver’s license.

A few parents discussed occupational disadvantages because of their children’s short stature (n=5; 15%). Parents reported worries about their children’s prospects in the labor market, and one mother worried about the disadvantages in work pay.

*“Where again, starting from me, you think it’s all okay now, but let the boy come into puberty, and he’s so small or he doesn’t get taller than 1.66 m. I think for a man, that’s already a problem. So many things. I have also informed myself a bit that there are studies in America that say that small men earn less in the same job, for example.”* (Mother of a 14- years-old (IGHD) and 12-year-old (ISS) adolescents)

Furthermore, one mother stated being worried about the growth development of her second child. The mother reported she is afraid that her daughter might also be short-statured and therefore controls the development of growth very intensively.

#### 3.2.7 Special needs

We coded statements about special needs into the category *special needs.* In this category, statements were coded when parents expressed their wishes and needs regarding how their child’s growth disorder should be handled. Statements mainly concerned the parent’s desire for a society more aware of the needs of short-statured people and their families.

Many interviewees reported intense wishes that other people would not treat their children differently than their peers (n=16; 48%). Even if their children are shorter than others, parents perceived the special treatment of short-statured children by the social environment as unhelpful and made them feel discontented. Several parents mentioned they would be happier if their children would be treated like any other child.

*“[ … ]. If you don’t say anything to him, there is no problem. I think that would be the very best if you treat him just like everyone else, and that’s it.”* (Father of a 15-year-old adolescent, ISS)

Some parents said they wished that pediatricians, teachers, pedagogues, and the industry would be more considerate of the needs of short-statured people and their families (n=8; 24%). For example, some mentioned that the industry should also produce smaller schoolbags because the normal ones were too big for short-statured children, which made it difficult to buy a suitable one. Some interviewees expressed being upset about how the pediatrician treated their child, so they mentioned the desire that pediatricians would be more aware of growth disorders and treat the children more age-appropriate.

Some parents wished for an exchange with other parents of short-statured children (n=4; 12%). They said it would help them better manage upcoming growth disorder issues. One mother said she would appreciate not being alone with short stature associated difficulties and challenges.

*“I mean, it is also an interesting topic. Above all, it’s nice when you’re not so alone with it. Otherwise, you always have no one to talk to. […].”* (Mother of 5-year-old identical twins, both, ISS)

A few parents suggested establishing a discussion group for parents of short-statured children, so they could exchange experiences with other parents of short-statured children and find solutions for problems together (n=4; 12%). Some said it would help them cope with the entire situation.

Moreover, some parents reported wishing their children would find ways to compensate for their growth disorder (n=4; 12%). For example, finding a peer group who accepts them how they are. Several interviewees stated that it would make them feel contented seeing their child getting accepted and integrated by other peers.

*“[…]. I would be very happy for him if he finally found someone who has exactly these character traits, where you say, okay, now you’ve found your partner.“* (Mother of a 13-year-old adolescent, ISS)

Other parents reported the idea of additional psychosocial support for their children when visiting the physicians for medical check-ups (n=6; 18%). Professional psychosocial support might ease coping with the health condition and its associated treatment or consequences. From the parents’ point of view, a connection to regular care would be associated with the advantage of an additional trusted person and reduced travel distances, relieving the parents.

Some parents talked about wanting their children to grow more (n=4; 12%). Parents said this would give them hope for the future and make them happy. Others felt the need to defend their children from bullies because they felt helpless. Furthermore, a few parents expressed the wish for more understanding and greater societal tolerance for growth disorders. They mentioned it would make life easier for them and their children if other people would approach them, for example, openly and without bias about the growth disorder.

#### 3.2.8 Individual resources

The category *individual resources* covers parental sources of strengths or resources in dealing with the child’s growth problems. Parents addressed straightforwardness about growth problems, optimism, or getting support as power sources.

Some interviewees reported that it helps them cope with their child’s chronic condition when dealing with this topic openly (n=7; 21%). For example, some expressed how it helps them to talk unconcealed about their child’s growth disorder with other people. One mother mentioned that being open-minded about this topic helped her find other parents with short-statured children.

*“And then I also deal with it quite openly and then also talk to people quite openly, because I think that is still a taboo subject with some people.”* (Mother of a 4-year-old child, IGHD)

Parents mentioned education about the growth disorder and support from their pediatrician and other family members as key factors in coping with their child’s condition (n=6; 18%). Parents stated they were more confident about the short stature when their pediatrician educated them about short stature and the therapy with hGH. Furthermore, parents reported that optimism about body growth helped both parents and their children deal with the issue.

*“But in the meantime, I’ve really learned to say: okay, it comes as it comes, I have to take it as it is. And when it’s good, it’s good, then we make the best of it. And if we have a bad phase, we also have to make the best of it. But it took me a long time, I have to be honest. It takes time to reach that point, you can’t do it overnight.”* (Mother of a 7-year-old child, ISS)

In addition, parents mentioned positive perceptions regarding the growth disorder by establishing an emotional distance from the topic of short stature (n=5; 15%). For example, parents stated it was relieving and helpful when adolescents were in charge of all the organizational issues, like executing the hGH injections themselves. A few parents mentioned that observing their children’s success in gaining height and physical development was helpful.

Parents reported feeling positive when their children succeed, for example, in sports (n=5, 15%). One mother reported being very proud that her son could qualify for a swimming competition, making it easier for her to deal with the short stature and its requirements in daily life.

## 4 Discussion

The parents in our sample reported experiencing a caregiving burden mainly due to the requirements of hGH treatment and mental stress due to their child’s growth disorder. Over half of the parents felt stressed about treatment with hGHs, mainly because they feared side effects or struggled with applying hGH via injections. The injections cause parental stress because parents struggle with causing their children pain, and organizational issues regarding the daily injections result in daily life challenges (37).

Many participants mentioned they adapted to the treatment application after some time but still had difficulties correctly interpreting the risks and side effects. They expressed being unsure where to search for valid information if not given by their pediatrician. Parents were concerned about the side effects of hGH treatment and reported hGH treatment having a big or extreme impact on their decision to seek medical treatment for their child’s short stature in a quantitative cross-sectional study (38). Accordingly, responsible healthcare providers need to educate their patients’ parents about possible side effects of hGH treatment and advise them on where to find evidence-based information. This professional support seems even more critical as the adherence of short-statured children and, therefore, the effectiveness of children’s treatment mainly depends on the parent’s psychological well-being and attitudes towards treatment (17). Knowing about treatment details could also help parents adjust their life to hGH treatment, as the daily injections often interfere with daily life routines (39).

Parental mental stress mainly results from frustration because of the reactions and behavior of the social environments toward their children (40). Additionally, parents were relieved when they received the children’s final diagnosis, emphasizing the great pressure parents experience by uncertainties, waiting times, and the diagnostic process. When comparing the anxiety levels of mothers of short-statured children with an IGHD diagnosis and mothers of children without a diagnosis, mothers of undiagnosed children report significantly higher anxiety levels (17). Waiting for the disclosure of a final diagnosis results in parental anxiety and concerns (37). These findings suggest that parents could benefit from psychological support during their children’s diagnostic process (17), and professional accompaniment, especially when no diagnosis can be made.

Our results confirm earlier findings on parents’ multiple caregiving burdens, stress, and impaired HRQOL in the care of IGHD/ISS children (17, 30, 37, 41, 42). The associations between children’s chronic health conditions and children’s and whole families’ well-being need to be considered in the healthcare and treatment of short-statured children (26, 29–31, 43).

Parents of male children/adolescents reported more problems and mental burdens due to the societal expectations of boys/men being tall. Parental worries regarding the additional challenges of short-statured males (44, 45) might result from societal expectations and norm orientations. From the parents’ perspective, society and the social environment emphasize standards, making short-statured children and their parents feel excluded. Getting stigmatized and bullied because their children failed to grow was demanding for parents. Stigmatization is often associated with physical and mental stress for the bullied children and their parents (46, 47).

Additionally, the distribution of parents of male and female children is very uneven, with a strong emphasis on boys. Although no gender differences were reported in the prevalence of short stature defined as a height below -2.25 SD, hGH treatment is more often indicated in males than females (48). Regardless of whether physicians indicate the more frequent indication for hGH treatment in boys or whether parents solicit this, it is clear that small body height is viewed as more negative for boys compared to age- and gender-adjusted norms. Parents of short-statured children reported being less able to accept the pathological short stature in boys than in girls (45). This may also be a reason for the disproportionate participation of parents of male children. A selection bias is also the exchange with other parents with similar experiences, which is perceived as support. This exchange with like-minded people is an essential resource for many parents, but also for affected children and adolescents, in dealing with the disease, the treatment, and the consequences in everyday life (49).

Impaired HRQOL of short-statured children’s parents due to stigmatization and other issues can cause additional problems. The positive link between parents’ stress levels and children’s well-being (50) highlights the need for parental support. Parents advise their children on coping strategies, serve as role models and help them adapt to their chronic health condition in the best possible way (51). So assuring parents can handle the caregiving burden will influence their children’s abilities to cope with their health-related burdens (52).

There is little research about parental HRQOL in the context of IGHD and ISS. The QoLISSY study group, an international research team, aimed to develop a patient-reported outcome measure to assess the children’s HRQOL and included two domains in the proxy-report focusing on the effects on parents and parents’ future worries (35). Parents mentioned future worries, especially in the context of their children’s perspective in the labor market (51, 53). Brodt et al. (40, 42) focussed on the burden of hGH treatment from the child’s perspective and the impact of the children’s treatment on the parents. In qualitative telephone interviews, parents reported their emotional impacts from their children’s IGHD; including worry for their child, anger or frustration over the reactions of others about their child’s height, relief when receiving a final diagnosis, and pressure in managing treatment for their children (40).

Several parents wished for parent support groups with other parents of short-statured children. Expansion and reinforcement of societal acceptance of diversity are required to meet the parents’ (and children’s) desire for regular care, including age-appropriate in contrast to height-appropriate treatment in daily life. Problems within the families, like ashamed grandparents leading to conflicts and disappointment in the parents, only mainly arose when other people made an issue out of the children’s growth disorder. Therefore, education and tolerance are crucial to support families of short-statured children.

## 5 Limitations

Our results are based on a small national sample, making the results hardly generalizable for all parents of IGHD/ISS children. Furthermore, it needs to be considered that people who participate in the interviews may not be representative. Hence, the parents’ attitudes and viewpoints might differ from other parents of IGHD/ISS children. Mainly mothers participated in the focus group discussions, and fathers were underrepresented. This can be explained by the fact that mothers are more often the primary caregivers than fathers (39).

Furthermore, parents of short-statured boys participated more often than those of girls. We did not collect any clinical data, so we cannot consider the time between the final diagnosis and the interviews or the duration of hGH treatment. Similarly, we were unable to consider sociodemographic factors when analyzing the interviews. However, it can be assumed that the fact that only parents who spoke sufficient German were included represents a bias. The parental experiences of short stature in the context of culture and parental gender need to be considered in future studies.

## 6 Conclusion

The influence of the child’s growth disorder due to IGHD/ISS on the parents and the whole family should not be underestimated. For physicians, it is essential to understand the parents’ caregiving burden, stress, and individual resources to initiate psychosocial intervention or parent support groups, if needed, and provide support and evidence-based information for parents having difficulty with hGH treatment. Healthcare and medical treatment in pediatric endocrinology should be family-centered. The parents especially strongly influence their children’s well-being and adaptation to the growth disorder by serving as role models. Therefore, one aim in the care of short-statured children has to be the appropriate support of parents resulting in confident parenthood and thus promoting the healthy development of these children.

## Data availability statement

The datasets analyzed during the study are not available publicly. This was done to keep the interview transcripts confidential. Requests to access these datasets should be directed to s.witt@uke.de.

## Ethics statement

The original QoLISSY study was reviewed and approved by the regional ethics board (PV3184) before it started. A regional ethics board achieved an additional ethics approval for re-analyzing the data (LPEK-0579a).

## Author contributions

SW and JQ developed the study concept and the design. LL and SW developed the coding guideline and coded the interviews. LL wrote the first draft of the manuscript. SW critically reviewed and revised the first draft for important intellectual content. All authors have critically revised subsequent drafts of the manuscript. All authors contributed to the article and approved the submitted version.
